# Beyond idiopathic short stature: refining the dialogue on nomenclature: **Response to the Letter to the Editor:** Arguments against the proposed change of the diagnostic term “idiopathic short stature” into “healthy short stature”

**DOI:** 10.20945/2359-4292-2026-0112

**Published:** 2026-10-05

**Authors:** Paulo F. Collett-Solberg, Alexander A. L. Jorge, Margaret C. S. Boguszewski

**Affiliations:** 1 Disciplina de Endocrinologia, Departamento de Clínica Médica, Faculdade de Ciências Médicas da Universidade do Estado do Rio de Janeiro, Rio de Janeiro, RJ, Brasil; 2 Unidade de Endocrinologia Genética (LIM25), Divisão de Endocrinologia, Hospital das Clínicas da Faculdade de Medicina da Universidade de São Paulo, São Paulo, SP, Brasil; 3 Departamento de Pediatria, Universidade Federal do Paraná, Curitiba, PR, Brasil

Dear Editor,

We thank Professors Wit and Ranke for their thoughtful response and for placing our proposal within the historical context of idiopathic short stature (ISS). Their contributions were fundamental to standardizing the definition of ISS and replacing the heterogeneous terminology that preceded it (^1^-^3^).

We respectfully suggest that the distinction between ISS and Healthy Short Stature (HSS) lies not in the certainty of our etiological knowledge, but in the clinical framework used to describe the child and communicate this status to families (^4^). ISS emphasizes the absence of an identified cause and is therefore inherently dependent on the diagnostic knowledge and technologies available at a given time. HSS, in contrast, describes the child’s current clinical status after appropriate evaluation and may remain applicable over time as long as the child continues to fulfill its defining criteria. The term “healthy” is not necessarily inconsistent with the broader WHO concept of health: short stature alone does not imply impaired physical, mental, or social well-being, particularly after careful and comprehensive clinical evaluation has failed to identify an underlying pathological condition (^5^). Importantly, HSS does not imply diagnostic certainty or the absence of future clinical surveillance; it remains compatible with genetic testing when indicated and with periodic reassessment throughout growth.

Our argument is that the ISS category is conceptually unstable: every etiological discovery - including those highlighted in the recently published international guideline on genetic testing of children with short stature (^6^) - progressively narrows the category and highlights its inherently provisional nature. And the change in category may affect reimbursement. As the authors anticipate, when polygenic scores and epigenetic tools become clinically available, more children will leave the ISS category for new diagnostic labels. “Idiopathic” is therefore an inherently provisional label that becomes obsolete with each discovery, whereas a descriptive term based on the child’s current clinical status remains valid.

We thank the authors for clarifying that “isolated short stature” is a descriptive starting point for further testing rather than a diagnostic category; we referred to it as proposed in the literature, for the semantic convenience of preserving the acronym, and did not propose it as a replacement for ISS.

Regarding the psychosocial rationale, which we consider central, recent data indicate that children with ISS experience increased psychosocial distress: in a Danish cohort of 16,121 children, ISS was associated with higher odds of bullying, loneliness, and insecurity (^7^). A recent review concluded that evidence that height augmentation directly improves psychosocial well-being is inconsistent, and that psychosocial factors may be more relevant than height gain (^8^). These findings support our view that terminology has consequences for families, and that avoiding a disease label for the lower extreme of normal height variation may reduce stigma without diminishing clinical vigilance (^9^).

We acknowledge the authors’ concerns that families might interpret “healthy” as an invitation to abandon follow-up. The term alone does not determine follow-up behavior; communication does. HSS is proposed as part of a framework that explicitly includes periodic reassessment and continued vigilance for red flags.

We remain convinced that terminology should pursue two objectives simultaneously: not to medicalize or stigmatize the lower extreme of normal height variation, and never to diminish clinical vigilance for pathological growth. We do not expect HSS to be the final word, and we are grateful to Professors Wit and Ranke for advancing this debate. We hope continued dialogue will lead to a terminology that is clinically precise, enduring, and humane.

## Data Availability

datasets related to this article will be available upon request to the corresponding author.
